# Is looped nasogastric tube feeding more effective than conventional nasogastric tube feeding for dysphagia in acute stroke?

**DOI:** 10.1186/1745-6215-8-19

**Published:** 2007-08-03

**Authors:** Jessica R Beavan, Simon Conroy, Jo Leonardi-Bee, Tim Bowling, Catherine Gaynor, John Gladman, Dawn Good, Peter Gorman, Rowan Harwood, Jan Riley, Tracey Sach, Wayne Sunman

**Affiliations:** 1 Nottingham University School of Community Health Sciences, B Floor, Medical School, Queen's Medical Centre, Nottingham, NG7 2UH, UK; 2 Queen's Medical Centre, Nottingham University Hospitals Trust, Derby Road, Nottingham, NG7 2UH, UK; 3 Nottingham City Hospital Campus, Nottingham University Hospitals Trust, Hucknall Road, Nottingham, NG5 1PB, UK; 4 Derbyshire Royal Infirmary, London Road, Derby, DE1 2QY, UK

## Abstract

**Background:**

Dysphagia occurs in up to 50% of patients admitted to hospital with acute strokes with up to 27% remaining by seven days. Up to 8% continue to have swallowing problems six months after their stroke with 1.7% still requiring enteral feeding. Nasogastric tubes (NGT) are the most commonly used method for providing enteral nutrition in early stroke, however they are easily and frequently removed leading to inadequate nutrition, early PEG (Percutaneous Endoscopic Gastrostomy) insertion or abandoning of feeding attempts. Looped nasogastric tube feeding may improve the delivery of nutrition to such patients.

**Methods:**

Three centre, two arm randomised controlled trial, with 50 participants in each arm comparing loop (the intervention) versus conventional nasogastric tube feeding. The primary outcome measure is proportion of intended feed delivered in the first 2 weeks. The study is designed to show a mean increase of feed delivery of 16% in the intervention group as compared with the control group, with 90% power at a 5% significance level. Secondary outcomes are treatment failures, mean volume of feed received, adverse events, cost-effectiveness, number of chest x-rays, number of nasogastric tubes and tolerability.

**Trial Registration:**

ISRCTN Number: ISRCTN61174381

## Background

Dysphagia occurs in up to 50% of patients admitted to hospital with hemispheric stroke [1-4]; up to 27% remain at risk of aspiration by seven days, and up to 8% have swallowing problems six months after their stroke [5] with 1.7% still requiring tube feeding [6]. Nutritional supplementation can reduce mortality in older people at risk of malnutrition, although this has not been shown specifically in the context of stroke [7]. During the period when patients with stroke are unable to take their full dietary requirements normally, the delivery of a liquid feed through a fine bore nasogastric tube is commonly used. The tube is usually secured with adhesive tape around the tube and to the patients face, and is often hooked behind the ear, where more adhesive tape may be used.

Unfortunately nasogastric tubes are frequently inadvertently dislodged, due to confusion, restlessness, communication and attention disorders during handling or normal movement. Partial dislodgement may leave the tube misplaced in the lungs, leading to a risk of aspiration. Dislodgement means that the tube needs to be re-sited, possibly causing distress and discomfort to the patient. Re-siting takes up nursing staff time, and may require a chest x-ray to confirm that the tip of the tube is in the stomach (especially in light of recent guidance [8]), adding further costs and inconvenience. With multiple re-sitings, and the associated delays, the amount of food that is delivered by conventional nasogastric tube may be significantly below the intended amount [9,10]. This gives rise to a risk of malnutrition, which is associated with poor outcomes [11-13]. In view of these difficulties, percutaneous endoscopic gastrostomies are considered but the results from the FOOD trial suggest that early PEG feeding may be injurious [14]. It would be helpful to find a simple and feasible means of using nasogastric tube feeding in acutely dysphagic stroke patients that reduces the likelihood of inadvertent nasogastric tube dislodgment.

Looped nasogastric feeding is a novel technique, early reports of which suggest that it allows more secure placement of a NG tube, but which is completely and easily reversible [15]. In this technique, the end result is that a loop is secured around the nasal septum; this loop is used to secure the nasogastric tube in place. This technique may result in considerably less tube dislodgement and more reliable feeding without compromising patient acceptability.

## Methods/Design

### Study design and participants

#### Study design

Three centre, two arm randomised controlled trial (Queens Medical Centre, Nottingham; Nottingham City Hospital, Derbyshire Royal Infirmary).

#### Subject definition

Any adult (> 18 years of age) with an acute clinically diagnosed stroke as defined by WHO standards ('A focal or at times global neurological impairment of sudden onset and lasting more than 24 hours, (or leading to death) and of presumed vascular origin') [16]; managed on the Stroke Unit. A clinical decision to attempt nasogastric tube feeding according to usual protocols has been made by the attending clinical team.

#### Exclusion criteria

Those not consenting to either NGT placement or to entry into the trial. Those for whom NG feeding is determined not to be in their best interests. Pregnant women. Those with contraindications to NG feeding (nasal trauma/malignancies).

#### Interventions

The research fellow and specialist stroke nurses will be trained in siting the looped nasogastric tube (LNGT) as well as conventional nasogastric tubes and will in turn train the ward based nurses in the same techniques.

#### Product details

The nasal loop is manufactured by Applied Medical Technology Inc (United States) and distributed in the UK by Pro-Care Ltd (UK). The CE mark is 93/42/EEC and the product has been through the FDA approval process in the United States.

#### Intervention group

The intervention group will receive all usual care except that the looped nasogastric feeding tube will be used for feed delivery. Subjects will have the loop component of the NGT sited as per manufacturer's instructions. The loop will be sited by either the research fellow, stroke nurses or ward staff who will have been fully trained in placing the loop. A nasogastric tube (NGT) will be passed and once in place fixed using the loop, thus creating the looped nasogastric tube. Upon confirmation that the NGT is correctly located, feeding will be commenced on an incremental fashion as per local protocols, which will vary between the centres

#### Control group

Participants will have a conventional nasogastric tube (CNGT) sited either by trained ward staff or by the study nurse. Upon confirmation that the NGT is correctly located, feeding will be commenced on an incremental fashion as per local protocols.

### Hypotheses

This trial will assess feed and fluid delivery by nasogastric tube; it does not seek primarily to assess nutritional outcomes, functional improvement or mortality. There are no well validated techniques for measuring change in nutritional outcomes, especially in the context of stroke. Anthropometric measures are difficult to obtain, because of immobility and inability to stand and so measure height. Biochemical indices (albumin, haemoglobin) are liable to confounding because of intercurrent infection and frequent comorbidity. Weight at baseline and two weeks will be measured and where possible demispan, allowing calculation of the Demiquet index [17] in order to provide a measure of nutritional status. It is unlikely that this trial will be able to show an improvement in function or nutrition, as the power requirements would require a number of participants an order of magnitude greater than anticipated; similar arguments can be applied to mortality.

The outcome measures are confined to the first two weeks following the onset of stroke, as after two weeks, there is the risk of contamination as individuals are offered PEG tubes as part of usual practice. For the purposes of this trial, PEG use within two weeks is considered as a failure of nasogastric feeding.

#### Primary hypothesis

Does use of the looped nasogastric tube in dysphagic acute stroke patients result in a greater proportion of nutritional prescription received per patient over a two week period than conventional nasogastric tube use?

#### Secondary hypotheses

Does use of the looped nasogastric tube in dysphagic acute stroke patients reduce the number of treatment failures during nasogastric feeding?

Does the use of the looped nasogastric tube enable greater total delivery of artificial nutrition?

What is the frequency of adverse events using this technique?

Is the looped nasogastric tube cost-effective compared to conventional nasogastric feeding, from an NHS perspective?

Does this technique reduce the proportion of patients requiring early PEG insertions?

Does looped nasogastric feeding improve nutritional indices compared to conventional nasogastric feeding?

Is loop feeding acceptable to patients?

### Sample size

Pilot information shows that conventional nasogastric feeding delivers approximately 43.5% of intended food (n = 4) [18] though based on small numbers, this audit data is consistent with previously published figures for NG feed delivery [10]. We estimate that an improvement to less than 60% would not be clinically worthwhile. No previous studies have been performed to assess the percentage of intended food that is delivered by a looped nasogastric tube in a comparable population. Therefore, we have assumed a conservative estimate for the common standard deviation, which relates to an effect size of 0.660.

A sample size of 50 in each group will have 90% power to be able to detect a 16.5% difference in the proportion of intended feed delivered between the conventional and looped nasogastric tube groups within two weeks assuming that the common standard deviation is 25%^1 ^using an independent two sample t test with a 5% two-sided significance level. Allowing for dropouts (10%), we aim to recruit a total sample size of 110 patients.

### Randomisation and blinding

Participants will be randomised with equal probability to usual care or the looped nasogastric technique by a computer generated pseudo-random list using random permuted blocks of randomly varying size, created by the Nottingham Clinical Trials Support Unit (CTSU) in accordance with their standard operating procedures and held on a secure server. The randomisation will be stratified by centre and Oxford stroke classification (TACS versus non-TACS). TACS describes the most severe form of stroke, with 90% of survivors either dead or heavily dependent at one year. The outcome for individuals with a non-TACS stroke is more favourable.

Access to the sequence will be confined to the CTSU Data Manager. Investigators will access the treatment allocation for each subject by means of a remote, internet-based randomisation system developed and maintained by the Nottingham CTSU. The sequence of treatment allocations will be concealed until interventions have all been assigned and recruitment, data collection, and statistical analyses are complete.

It will not be possible to blind participants nor the data collectors to the treatment allocation, due to the nature of the intervention. However, all outcomes will be assessed by a blinded assessor and analysis will be blind to the treatment allocation.

### Procedures and observations

#### Consent

Verbal and/or written consent will be obtained from all participants where possible. Where dysphasia or other barriers exist to obtaining verbal consent, if the clinicians affirm that it is in the patient's best interest to participate (at risk of minimal harm from the intervention), assent will be sought from the next of kin, mirroring clinical practice where consent to nasogastric feeding is established. Information sheets will be provided to participants and family members. Individuals who are entered into the trial through a process of assent, will be reviewed regularly and given the opportunity for fully informed consent. They may choose to withdraw from the study at any stage without compromising their care. Consent processes are based on Department of Health and MRC Guidance [19].

### Patients not recruited into the study

Patients not recruited into the study will receive all usual care, but will not be eligible for the looped nasogastric tube, which is not standard therapy.

### Baseline measurements

Age, sex, hospital, previous stroke, residential status, Oxford stroke classification, NIHSS (National Institute of Health Stroke Scale), time from stroke onset, Glasgow Coma Scale score at 24 hours, weight and demi-span, and whether treated for chest infection between admission and allocation.

### Outcome measurements

#### Definition of primary outcome

Primary outcome: percentage of nutritional prescription received (amount delivered/amount intended as per dietician's prescription, including all feed and fluids) delivered in the two weeks from allocation or at the point NG feeding is stopped earlier on clinical grounds.

#### Secondary outcomes

• Number of times tube re-sited in two weeks; treatment failure/completed treatment as specified (where treatment failure means any occasion where attempts at nasogastric tube feeding is ceased before normal oral intake is established, and includes multiple failed attempts at passing a tube, use of a PEG (in first two weeks), death or deterioration such that feeding is considered unsafe or unwanted)

• Mean volume of nasogastric feed delivered in the two weeks from allocation

• Proportion of patients requiring early PEG insertions

• The technical efficiency (that is whether the best outcome is being achieved within a given set of resources) of looped nasogastric feeding after stroke compared to ordinary nasogastric tubes will be assessed from an NHS perspective to see if this new technology offers value for money. An intervention specific outcome will be used to estimate an incremental cost-effectiveness ratio in the form of a cost per change in percentage nutritional prescription received.

• Change in Demiquet index from baseline to two weeks (weight in kilograms)

• Tolerability/acceptability of technique by questionnaires to patients, families and nursing staff.

### Adverse events

Tube related: reports of nasal irritation, complications associated with nasogastric tube use

Aspiration pneumonia

Diarrhoea

Gastrointestinal bleeding

Refeeding syndrome

### Follow up measures

We will follow up trial participants at three months; we will only perform descriptive statistics due to lack of power for formal statistical comparisons. These follow up measures will include functional outcomes, length of stay, feeding status, mortality and discharge placement. Tolerability at three months will be assessed using a postal questionnaire and supplemented by interview when necessary. This is important as perceptions of tube tolerability may be different in the recovery phase.

### Ascertainment of outcomes

Research Fellow and stroke nurses at each site will assist in the collection of data, as well as training ward staff in the technique of tube placement (including the looped tube).

#### Primary outcome

The volume of feed and fluid administered via the NGT will be recorded by ward nursing staff on a daily basis. The amount of feed delivered can be accurately recorded as feed delivery is regulated using digital pumps. The pumps enable accurate recording of the amount of feed delivered (or not delivered) in millilitres. This information will be collated by the Research Fellow when they visit the stroke unit each day.

#### Secondary outcomes

Tube usage and tube related complications will be monitored and the data collated using, to be completed for each patient every 24 hours. The form will be completed by ward staff with the support of the research fellow.

Treatment failure/completed treatment (where treatment failure means any occasion where attempts at nasogastric tube feeding is ceased before normal oral intake is established, and includes multiple failed attempts at passing a tube, use of a PEG (in the first two weeks), death or deterioration such that feeding is considered unsafe or unwanted) will also be recorded on this form. In such cases, the circumstances will be abstracted from the medical notes (< 1/2 side A4), without reference to treatment group. The abstract will then be anonymised and e-mailed to a medically qualified blinded assessor (RH). The assessor will then assign the outcome as failure/no failure (e.g. when feeding stopped because of return of oral feeding).

Weight (kilograms) and demispan (in cm: using stainless steel tape measure, finger roots to sternal notch), will be measured at baseline and at two weeks.

Both groups will be asked to complete a tolerability questionnaire regarding NG feeding, this will be adapted for dysphasic patients.

### Adverse events

Tube related adverse events will be monitored on a daily basis, by ward nursing staff and the clinical fellow. Any reports of nasal irritation, bleeding or discomfort will be recorded.

Diarrhoea will be recorded using standard ward documentation (stool charts)

For the purposes of the study, aspiration pneumonia is defined as the clinical diagnosis appearing in the medical notes associated with a prescription of appropriate antibiotics. As with treatment failure/treatment complete, case note abstraction will be used and the abstract sent to a blinded assessor (JG). These data will be recorded by the research fellow.

### Follow up measures

At three months, the research fellow will check the medical records and/or with the patient's GP to check the patient's status and will then confirm the discharge data, feeding status and collect the Barthel Activities of Daily Living score and Euro-QoL using a postal questionnaire. Non-responders will be prompted via telephone 2 weeks after the questionnaire has been sent and a home visit arranged if necessary. Length of hospital stay (days), mortality, residential status at discharge (own home/own home with help/residential home/nursing home) will be recorded by the research fellow based on a review of medical records. Participants will also be asked to complete a questionnaire about the tolerability of the feeding method they received.

### Cost-effectiveness

Cost-effectiveness analysis will be performed from an NHS perspective only since in the trial period of two weeks it is unlikely to have any cost impacts on the patient or other services, such as social services. Components to be considered in the cost-effectiveness analyses will include:

• Cost of feed

• Feed wastage (number of bags of feed discarded due to incomplete delivery of feed; cost per bag is likely to vary between centres)

• Cost of nasogastric tube (NGT)

• Cost of looped NGT equipment

• Standardised costs based on sampling for: portering time (to and from x-ray), nursing time (siting/re-siting tubes), cost of PEG (equipment, placement, maintenance), cost of complications (aspiration pneumonia) and length of stay. We will also test for any differences in discharge destination, but the study is not powered to look at this specifically and it is not expected that any differences will be observed

• Cost of treating any incident refeeding syndrome (phosphate, potassium an magnesium supplements as well as treatment of the complications of refeeding syndrome, such as fluid retention and neuromuscular fatigue)

• Cost of training in siting looped NGTs will be derived from the pre-study preparation with the study nurses

• Resource use data will be derived from observation of a sample in order to estimate standard unit costs. For example, the cost of re-siting an NGT will be derived from observing the process and attaching relevant unit costs – products, time (nursing, medical, portering, and radiography) and the cost of X-rays. Standard costs will then be approximated. Other resource used will be collected from clinical notes and costed using published unit cost data [20,21]. The cost analysis results will be combined with data on mean volume of nasogastric feed in order to produce an incremental cost-effectiveness ratio of the cost per % change in mean volume of nasogastric feed.

## Discussion

This study began recruitment in September 2006 and will complete data collection by December 2007. The results of the study will allow physicians caring for stroke patients to make informed decisions about the best way to provide nutrition to patients with dysphagia in the early stages. The results may be applicable to other patients with dysphagia or requiring nutritional support.

## Approval Process

The study has been reviewed by Nottingham Research Ethics Committee and granted MREC approval.

## Funding

The study has been funded through a fellowship provided by the Royal College of Physicians/Dunhill Medical Trust.

## List of abbreviations

CNGT Conventional Nasogastric Tube

CTSU Clinical Trials Support Unit

FDA Food and Drug Administration

GP General Practitioner

LNGT Looped Nasogastric Tube

MRC Medical Research Council

NGT Nasogastric tube

NIHSS National Institutes of Health Stroke Scale

PEG Percutaneous Endoscopic Gastrostomy

TACS Total Anterior Circulation Stroke

WHO World Health Organisation

## Competing interests

The author(s) declare that they have no competing interests.

## Authors' contributions

SC wrote the main protocol, which was contributed to by JG, JLB and JB. The whole study group reviewed and approved the protocol.
